# α-Synuclein seeding amplification assays for diagnosing synucleinopathies: an innovative tool in clinical implementation

**DOI:** 10.1186/s40035-024-00449-2

**Published:** 2024-11-21

**Authors:** Yaoyun Kuang, Hengxu Mao, Xiaoyun Huang, Minshan Chen, Wei Dai, Tingting Gan, Jiaqi Wang, Hui Sun, Hao Lin, Qin Liu, Xinling Yang, Ping-Yi Xu

**Affiliations:** 1 Department of Neurology, The First Affiliated Hospital of Guangzhou Medical University, Guangzhou, 510120 China; 2 Houjie Hospital of Dongguan, Dongguan, 523000 China; 3 Department of Neurology, Xinjiang Uygur Autonomous Region People’s Hospital, Urumqi, 830054 Xinjiang China; 4 The Second Affiliated Hospital of Xinjiang Medical University, Urumqi, 830054 Xinjiang China

**Keywords:** α-Synuclein, Movement disorders, Seed amplification assay, Quiescent seed amplification assay, Diagnosis

## Abstract

The spectrum of synucleinopathies, including Parkinson’s disease (PD), multiple system atrophy (MSA), and dementia with Lewy bodies (DLB), is characterized by α-synuclein (αSyn) pathology, which serves as the definitive diagnostic marker. However, current diagnostic methods primarily rely on motor symptoms that manifest years after the initial neuropathological changes, thereby delaying potential treatment. The symptomatic overlap between PD and MSA further complicates the diagnosis, highlighting the need for precise and differential diagnostic methods for these overlapping neurodegenerative diseases. αSyn misfolding and aggregation occur before clinical symptoms appear, suggesting that detection of pathological αSyn could enable early molecular diagnosis of synucleinopathies. Recent advances in seed amplification assay (SAA) offer a tool for detecting neurodegenerative diseases by identifying αSyn misfolding in fluid and tissue samples, even at preclinical stages. Extensive research has validated the effectiveness and reproducibility of SAAs for diagnosing synucleinopathies, with ongoing efforts focusing on optimizing conditions for detecting pathological αSyn in more accessible samples and identifying specific αSyn species to differentiate between various synucleinopathies. This review offers a thorough overview of SAA technology, exploring its applications for diagnosing synucleinopathies, addressing the current challenges, and outlining future directions for its clinical use.

## Introduction

Synucleinopathies are a diverse group of proteinopathies characterized by the accumulation of intracellular αSyn aggregates [1]. Lewy body diseases (LBDs) and multiple system atrophy (MSA) are two main categories of disease within this group [2]. LBDs include a spectrum of neurodegenerative disorders, such as Parkinson's disease (PD), PD with dementia (PDD), and dementia with Lewy bodies (DLB). MSA, on the other hand, has two primary clinical subtypes: MSA with predominant cerebellar ataxia (MSA-C) and MSA with predominant parkinsonism (MSA-P).

As the most common synucleinopathy, PD is diagnosed based on clinical motor symptoms, accompanied by brain imaging as a supportive tool [3]. Notably, the motor symptoms of PD typically appear after significant neuronal degeneration, when 50%–80% of nigral dopaminergic neurons have been lost [4]. This means that PD is often diagnosed in its later stages when both motor and non-motor symptoms are already present. Emerging evidence indicates that various symptoms, such as essential tremor (ET), olfactory dysfunction, sleep disturbances, depression, anxiety, constipation, and other signs of autonomic dysfunction, may appear before the classic motor symptoms of PD [5].

DLB diagnosis depends on key features that overlap with PDD, including cognitive decline, parkinsonism, fluctuating cognition and alertness, and visual hallucinations. A critical factor in distinguishing DLB from PDD is the timing of dementia onset relative to parkinsonism. If dementia occurs before, concurrently with, or within one year of parkinsonism onset, then a DLB diagnosis will be made [6]. If dementia occurs after one year, then a PDD diagnosis will be made [7]. REM sleep behavior disorder (RBD) is now also recognized as a core feature of DLB [8]. However, distinguishing between synucleinopathies in the early stages can be difficult due to their highly varied clinical presentations.

Neuropathologically, PD and DLB are characterized by αSyn aggregates forming Lewy bodies and Lewy neurites in neurons and axonal processes [9], while MSA is characterized by αSyn inclusions in oligodendroglia [10]. These aggregates may disrupt normal neuronal function and contribute to neurological decline. However, the presence of Lewy pathology is neither necessary nor sufficient for a PD diagnosis, as some PD patients do not exhibit these features. For example, Lewy bodies can be found in conditions unrelated to PD, such as mitochondrial membrane protein-associated neurodegeneration, and may be absent from clinical cases of PD, including those associated with *LRRK2* or Parkin mutations [11]. Moreover, Lewy bodies are not exclusive to PD. Some patients with PD lack neocortical Lewy bodies, while others with Lewy bodies may not have PD [12, 13]. These complexities have prompted ongoing discussions among specialists regarding the challenges and future directions in synucleinopathy research, particularly in understanding their molecular pathogenesis. This has led to new approaches to classifying and diagnosing PD from a biological perspective. Recently, two groups of scientists have introduced new ontologies for PD and related disorders: the Neuronal αSyn Disease Integrated Staging System (NSD-ISS) and the SynNeurGe criteria [14, 15]. Both frameworks aim to categorize disease subtypes, including at the early stages before clinical appearance of parkinsonism, using SAA to detect misfolded αSyn with high sensitivity.

The detection of αSyn, particularly via SAA, holds promise for earlier and accurate diagnosis of synucleinopathies. However, there are still challenges to be addressed, including the need for extensive validation to ensure accuracy, the ethical considerations regarding early diagnosis in the absence of curative treatments, and the complexities of interpreting results at different stages of the disease. Though progress has been made in improving the sensitivity and specificity of the tests, standardizing the assays across laboratories and evaluating its effectiveness in preclinical stages remain crucial. Over time, with more data gathered by multiple laboratories, these challenges may be resolved, paving the way for more reliable clinical application of αSyn detection.

## αSyn physiology and pathology

αSyn is encoded by the *SNCA* gene on chromosome 4 (4q22.1), and consists of 140 amino acids with a molecular mass of approximately 15 kDa [16]. It is structured into three main domains: a C-terminal region rich in acidic residues, a central non-amyloid component (NAC) region that promotes oligomerization and aggregation due to its hydrophobic property, and an N-terminal region containing four 11-residue imperfect repeats with a KTKGEV consensus sequence, which supports lipid binding [17].

Under normal physiological states, αSyn exists as an intrinsically disordered, soluble monomer distributed across several cellular locations, including synaptic terminals, the endoplasmic reticulum, Golgi apparatus, neuronal nuclei, mitochondria, and the endolysosomal system [17]. However, under certain experimental or disease-related conditions, it can undergo pathological transformations, where it self-assembles into amyloid aggregates. While the exact mechanisms that trigger αSyn oligomerization remain unclear, αSyn interaction with lipids is a key factor contributing to its pathological fibrillation.

Different lipids influence αSyn aggregation in varied ways. Some lipids facilitate the self-assembly of αSyn into fibrils, while others act as inhibitors [18–21]. The impact of phospholipids on αSyn aggregation is dependent on both the lipid type and the lipid-to-protein ratio. At specific ratios, some lipids can accelerate fibril formation by providing nucleation sites, which promote elongation [19]. However, when there are sufficient phospholipid membranes available for binding relative to the number of lipid-bound αSyn molecules, aggregation is inhibited, as the helical conformation of membrane-bound αSyn prevents fibril formation [22]. Moreover, αSyn binds to small unilamellar phospholipid vesicles containing acidic phospholipids, resulting in an increase of α-helicity from 3% to approximately 80%, thereby stabilizing its secondary structure [23]. Consistently, the V15A mutation of αSyn associated with familial PD leads to a reduced affinity of αSyn to phospholipids and increased propagation activity compared to the wild-type αSyn [24].

Recent studies indicate that αSyn has a strong affinity for lysophospholipids, particularly lysophosphatidylcholine [25]. This binding is significant because it prevents the pathological aggregation of αSyn, suggesting that some lipids can protect against fibril formation. Factors such as lipid oxidation and aging can further modulate lipid properties, affecting interactions of αSyn with membranes [21], leading to behavioral change of αSyn from being functional to being harmful. This suggests that the surrounding lipid environment plays a crucial role in αSyn’s propensity to form fibrils.

The cytotoxic effects of αSyn multimers, particularly oligomers, are closely associated with increased oxidative stress, impaired axonal transport, disruption of the ubiquitin–proteasome system, mitochondrial dysfunction, and synaptic dysfunction [26–28]. Moreover, the ability of αSyn to propagate between neurons through a mechanism known as “seeding” exacerbates these harmful effects [29]. In this prion-like process, pathological αSyn induces the misfolding and aggregation of soluble αSyn monomers, acting as “seeds” that template and propagate further aggregation. The evidence supporting this seeding mechanism is compelling. A key example came from experiments where αSyn preformed fibrils (PFFs)—synthetic analogs of αSyn fibrils—or αSyn aggregates derived from patient Lewy bodies were injected directly into the brains of wild-type mice. These injections successfully induced hallmark αSyn pathology in the recipient mice, resulting in the loss of dopaminergic neurons, neuroinflammation, and behavioral deficits similar to those seen in PD [30, 31].

αSyn phosphorylation at serine 129 (pS129) plays a complex and dual role. Under physiological conditions, pS129 is implicated in the regulation of the biological activity of αSyn, particularly activity in pathways associated with neuronal activity, thus contributing to the functioning of neurons [32, 33]. However, in the context of diseases, particularly neurodegenerative disorders like PD, pS129 phosphorylation becomes closely associated with αSyn aggregation and its involvement in disease progression [34]. While αSyn aggregation is a hallmark of disease, the precise relationship between pS129 and the aggregation process remains incompletely understood. Some studies, particularly those in rodent models, suggest that pS129 may enhance αSyn aggregation, potentially exacerbating the toxic effects on neuronal function [35]. Conversely, other research indicates that pS129 could play a protective role under certain conditions [36, 37]. It has been proposed that phosphorylation at serine 129 occurs following the initial deposition of αSyn aggregates, where it may function to limit further fibril propagation [38, 39]. This result posits that phosphorylation might not always contribute to the seeding capacity of αSyn—an essential step in the spread of pathology from one neuron to another. In this scenario, phosphorylated αSyn could act as a “brake” on the aggregation process, preventing the continuous seeding of fibrils and thereby slowing disease progression.

## The origin and transmission of αSyn pathology

αSyn is a protein abundantly expressed in the CNS [40]. Although pathological αSyn is predominantly found in the brain, increasing evidence suggests that in some patients, αSyn pathology may originate in peripheral organs before spreading to the brain [41]. This observation has led to the development of a dual transmission model of αSyn pathology, comprising the ‘brain-first’ and ‘body-first’ hypotheses [42, 43].

In the brain-first subtype, αSyn pathology originates within the CNS, typically beginning unilaterally in regions such as the amygdala [44]. This unilateral onset causes the pathology to spread primarily to the same side of the brain, including the substantia nigra, leading to asymmetric dopaminergic degeneration and motor symptoms that are more pronounced on one side of the body. In contrast, the body-first PD subtype suggests that αSyn pathology starts in the peripheral autonomic nervous system. Braak et al. demonstrated that synucleinopathy lesions could originate in the peripheral nervous system, particularly in the gut, and spread via the autonomic nerves to the dorsal motor nucleus of the vagus nerve to both sides of the brainstem [45–47]. This results in symmetric spread of αSyn within the CNS, leading to more balanced dopaminergic degeneration and less pronounced motor asymmetry. By the time of diagnosis, body-first patients typically have a more widespread, symmetric burden of pathology, which is associated with faster disease progression and more rapid cognitive decline.

Another origin theory, the dual-hit hypothesis, proposes that the initial Lewy pathology arises simultaneously in the olfactory bulb and the enteric nervous system (ENS) plexuses during the earliest stages of PD [48]. However, recent studies have indicated that the pathological process usually begins in either the olfactory bulb or the ENS, seldom affecting both simultaneously[49].

Once αSyn aggregates reach the brain, they can propagate to autonomic nerves and be transferred back to peripheral tissues that are rich in autonomic innervation [50, 51]. These processes allow the pathological forms of αSyn to move between neurons and across different regions, facilitating the dissemination of the aggregates throughout both central and peripheral tissues. For instance, αSyn pathology has been detected in peripheral nerves located in tissues such as skin and oral mucosa, indicating a pathological link between the autonomic nervous system and the CNS [52, 53]. This finding has important diagnostic implications, as the detection of pathological αSyn in skin biopsies or olfactory mucosal offers a potential method for identifying PD before significant neurodegeneration occurs [47, 54]. Beyond the nervous system, αSyn pathology also extends to neuroendocrine organs and glands. For example, phosphorylated αSyn has been found in the posterior lobe of the pituitary gland [55] and in the salivary glands [56]. Understanding these transmission pathways not only enhances our knowledge of PD progression but also opens new avenues for early detection and intervention.

## αSyn SAAs in readily available biological matrices

**Fig. 1:**
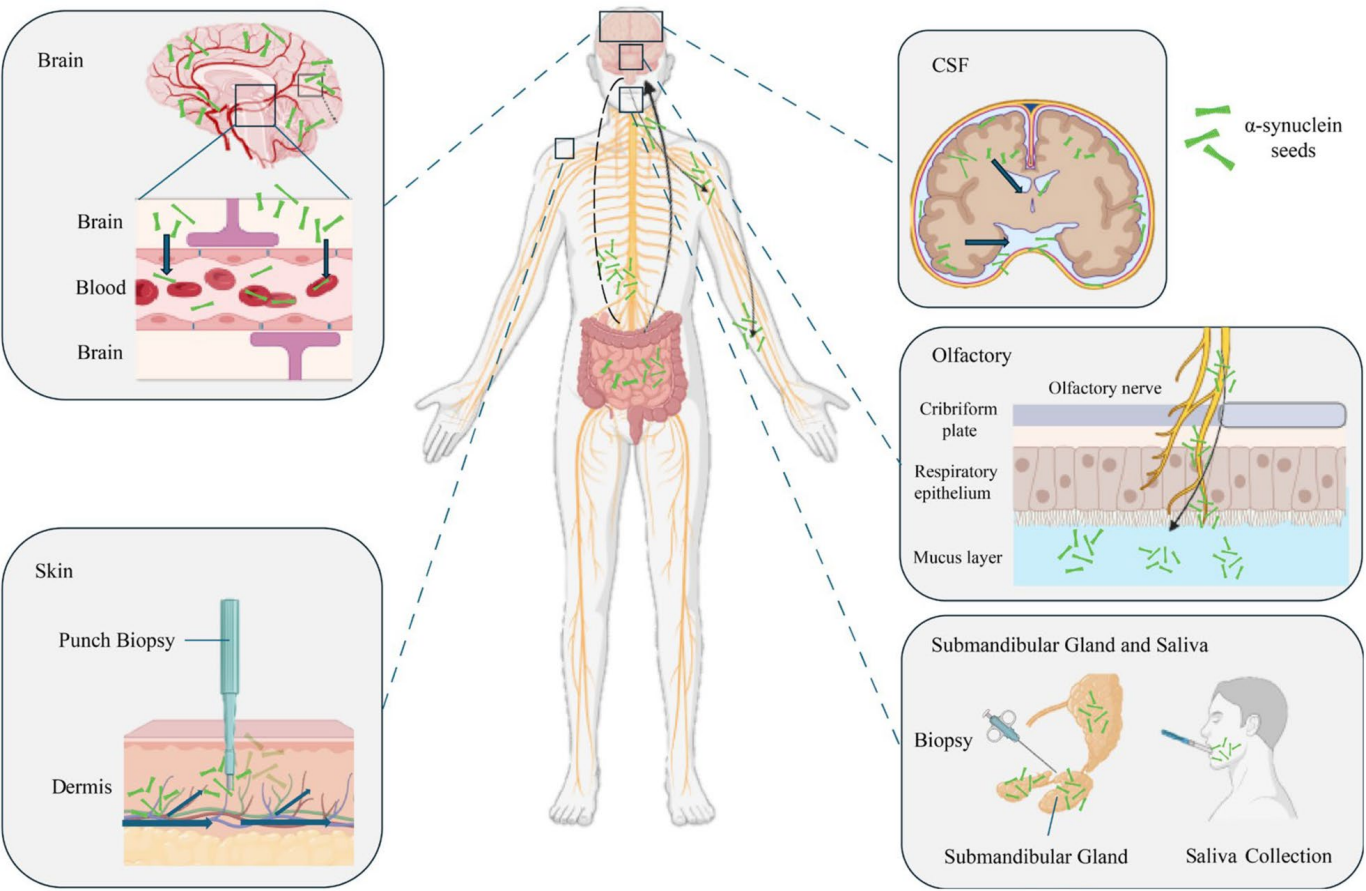
Schematic overview of the dissemination of pathological α-synuclein (αSyn) aggregates in various regions of the brain and peripheral tissues as well as in biological fluids. Graphic created with BioRender.com

**Fig. 2:**
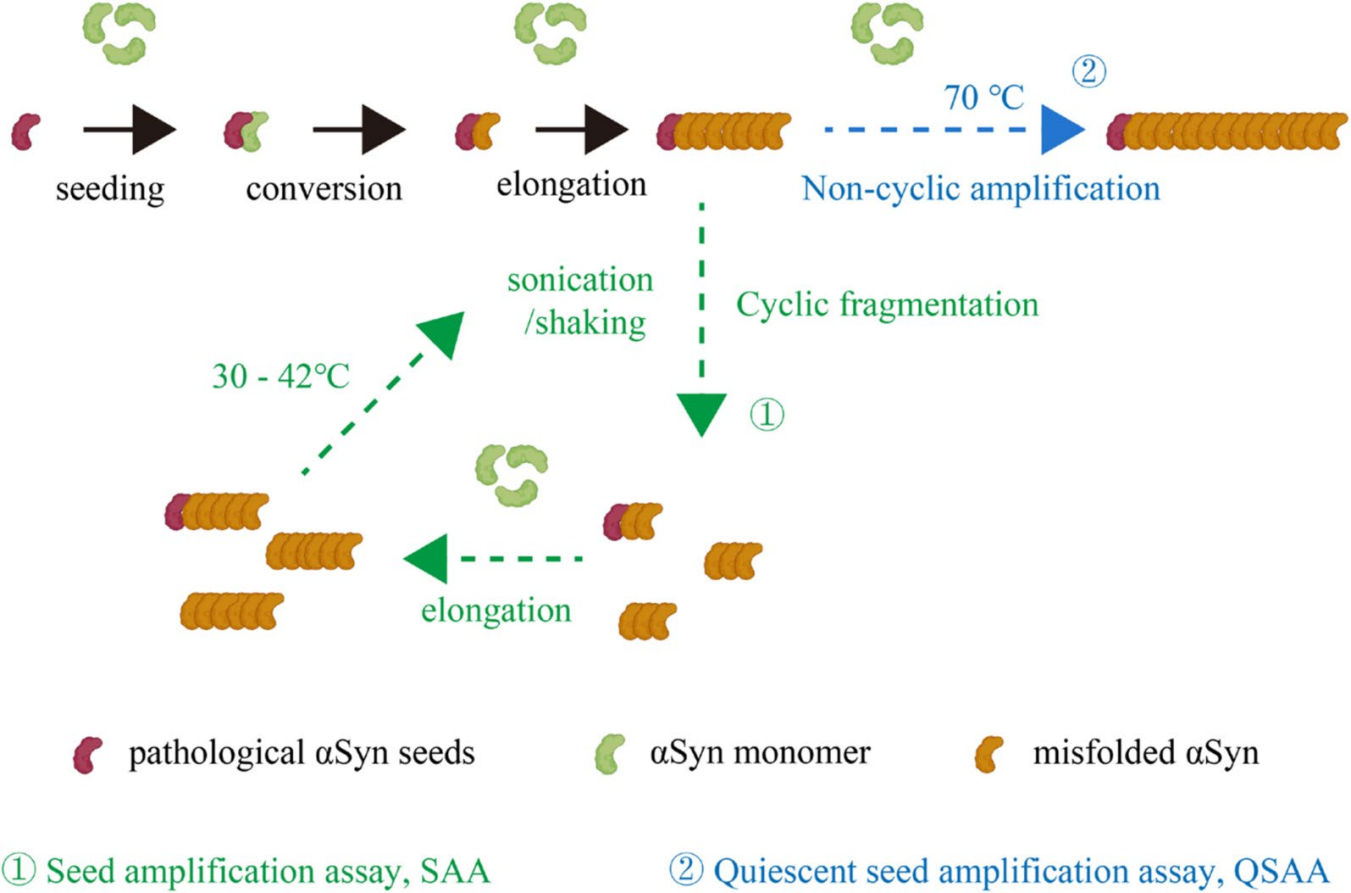
Mechanisms of seed amplification assays (SAA) and quiescent seed amplification assays (QSAA). Both assays induce misfolding of normal proteins into pathological forms, leading to fibril formation. The legend highlights the active fragmentation in SAA and the passive amplification approach of QSAA. Graphic created with BioRender

Fairfoul et al*.* were the first to use the protein amplification assays to detect misfolded αSyn in cerebrospinal fluid (CSF) [57]. Since then, these assays have been optimized to detect αSyn in olfactory mucosa, submandibular gland biopsies, blood, skin, and saliva of patients with PD and other synucleinopathies [58–65] (Fig. 1). Table 1 provides a summary of studies on αSyn SAA using different sample types. αSyn SAAs rely on the intrinsic self-replicative nature of misfolded αSyn aggregates (seeds) to multiply them using recombinant αSyn (rec-αSyn) in vitro. In these assays, αSyn seeds circulating in biological fluids and deposited in tissues are amplified by a cyclical process that includes aggregate fragmentation into smaller self-propagating seeds, followed by elongation at the expense of rec-αSyn (Fig. 2). Protein misfolding cyclic amplification (PMCA) and real-time quaking-induced conversion (RT-QuIC) are two key protein amplification assays for detecting misfolded αSyn seeds, both classified under the broader category of αSyn SAAs. Although RT-QuIC and PMCA are both powerful assays designed to detect misfolded αSyn seeds, they operate via distinct mechanisms and have different practical applications. Table 2 summarizes the similarities and differences between RT-QuIC and PMCA.

**Table 1:** Summary of studies on αSyn SAA using different sample types

Tissue Type	Sensitivity	Specificity	Study
Brain	10%–100%	50%–100%	Candelise et al., 2019 [136]
			Manne et al., 2019 [137]
			Poggiolini et al., 2021 [138]^*,#^
			Bentivenga et al., 2024 [139]
			Mao et al., 2024 [97]
Olfactory mucosa	44.4%–90%	75%–100%	De Luca et al., 2019 [140]
			Stefani et al., 2021 [54]
			Perra et al., 2021 [141]
			Bargar et al., 2021a [61]^*,#^
			Bongianni et al., 2022 [142]
Oral mucosa	67.30%	90.30%	Zheng et al., 2024 [59]^*,#^
Salivary	61.1%–86%	78%–94.4%	Luan et al., 2022 [126]^*^
			Vivacqua et al., 2023 [60]^**#**^
Serum	95%	92.20%	Okuzumi et al., 2023 [63]^*,#^
EVs	62%–99%	100.00%	Kluge et al., 2024a [72]^*,#^
			Kluge et al., 2024b [143]
Skin	75%–100%	80%–100%	Manne et al., 2020a [71]^*^
			Wang et al., 2020 [58]^**#**^
			Kuzkina et al., 2021 [68]
			Iranzo et al., 2023 [47]
			Kuang et al., 2024 [65]
			Mao et al., 2024 [97]
SMG	75%–93.75%	100%	Manne et al., 2020b [64]^*,#^
CSF	15.4%–100%	76.9%–100%	Fairfoul et al., 2016 [57]^**#**^
			Groveman et al., 2018 [128]^**#**^
			Bongianni et al., 2019 [144]
			Kang et al., 2019 [145]
			van Rumund et al., 2019 [146]
			Garrido et al., 2019 [147]
			Manne et al., 2019 [137]^*,#^
			Rossi et al., 2020 [148]^*^
			Orrù et al., 2021 [149]
			Bargar et al., 2021b [150]^**#**^
			Quadalti et al., 2021 [151]
			Iranzo et al., 2021 [152]^**#**^
			Brockmann et al., 2021 [153]
			Donadio et al., 2021 [70]^**#**^
			Russo et al., 2021 [107]
			Hall et al., 2022 [154]^*^
			Poggiolini et al., 2022 [155]^*^
			Compta et al., 2022 [156]^**#**^
			Majbour et al., 2022 [157]
			Concha-Marambio et al., 2023 [158]
			Brockmann et al., 2024 [159]
			Samudra et al., 2024 [160]
			Bellomo et al., 2024 [114]

Abbreviation: EVs, Extracellular vesicles; SMG, Submandibular glands; CSF, Cerebrospinal fluid; *, the highest sensitivity; #, the highest specificity

**Table 2:** Key differences and similarities between real-time quaking-induced conversion (RT-QuIC) and protein misfolding cyclic amplification (PMCA)

	RT-QuIC	PMCA
Purpose	Detecting misfolded αSyn	Detecting misfolded αSyn
Amplification mechanism	Physical shaking (quaking) to induce protein aggregation	Cycles of sonication and incubation to amplify aggregates
Substrate	Recombinant αSyn produced in vitro, highly purified	Either recombinant αSyn or tissue-derived αSyn (e.g., from brain samples)
Real-time detection	Yes. Real-time monitoring based on ThT fluorescence	No. Post-amplification detection such as immunoblotting is required
Sensitivity	High	Extremely high
Operational complexity	Simple and suitable for high-throughput and fast detection	Complex and time-consuming, often for research use
Time	Short, providing results rapidly	Longer, requiring more time for amplification and analysis
Clinical application	Common in clinical diagnostics, fast and efficient	Less commonly used in clinical settings, mainly for research purpose
Safety	Simple and safe, with lower biological hazard	More complex handling with additional experimental steps

αSyn SAAs in CSF have demonstrated high accuracy for differentiating LBD from other conditions unrelated to misfolded αSyn [57, 66]. However, due to the intrinsic limitations, such as the need for lumbar puncture, researchers are exploring more accessible biological matrices like skin and blood to detect αSyn pathology. Skin biopsy, a minimally invasive procedure, has demonstrated comparable diagnostic performance to CSF in distinguishing PD patients from non-PD controls [67–69]. Notably, results can be obtained within less than 24 h. This rapid and accurate detection makes skin αSyn SAA a promising peripheral biomarker for synucleinopathies [58, 69–71]. In these protocols, a threshold is established; a fluorescence signal exceeding the threshold indicates the presence of detectable amyloid fibrils. The ability to reliably and efficiently detect pathological αSyn in the skin makes it a reliable peripheral marker for synucleinopathies.

Blood-based αSyn detection, specifically through serum SAA and neuronal extracellular vesicles (EVs), has also gained attention. Serum SAAs, using an immunoprecipitation-based method (IP/SAA), have proven capable of identifying pathogenic αSyn in individuals with synucleinopathies and distinguishing PD and MSA patients from controls [63]. Furthermore, neuronal-derived αSyn extracted from EVs in blood plasma has shown the potential to predict PD risk and detect misfolded αSyn years before clinical diagnosis [62, 72, 73]. Additionally, a longer disease duration has been linked to decreased αSyn seeding activity in PD, as identified by neuronal EVs in the blood [74]. Another notable finding is the high concentration of αSyn in red blood cells (RBCs) [75]. Moreover, αSyn is also abundantly expressed in various other cell types within the hematopoietic system, such as T and B lymphocytes, monocytes, natural killer (NK) cells, and megakaryocytes [76, 77]. This widespread expression indicates that αSyn plays an essential role in the development and functioning of hematopoietic cells. Studies in αSyn-deficient mouse models further support this, as the absence of αSyn results in dysfunctional hematopoietic cells, highlighting its critical role in cell maturation [78–80]. Therefore, the high levels of αSyn found in RBCs likely stem from its expression during earlier stages of hematopoiesis before the cells lose their nuclei. Research has shown that hemoglobin-binding αSyn (Hb-αSyn) levels are elevated in patients with PD and MSA, and αSyn accumulation in the aging brain correlates with an increase in the Hb-αSyn complex in RBCs [81–84].

**Fig. 3:**
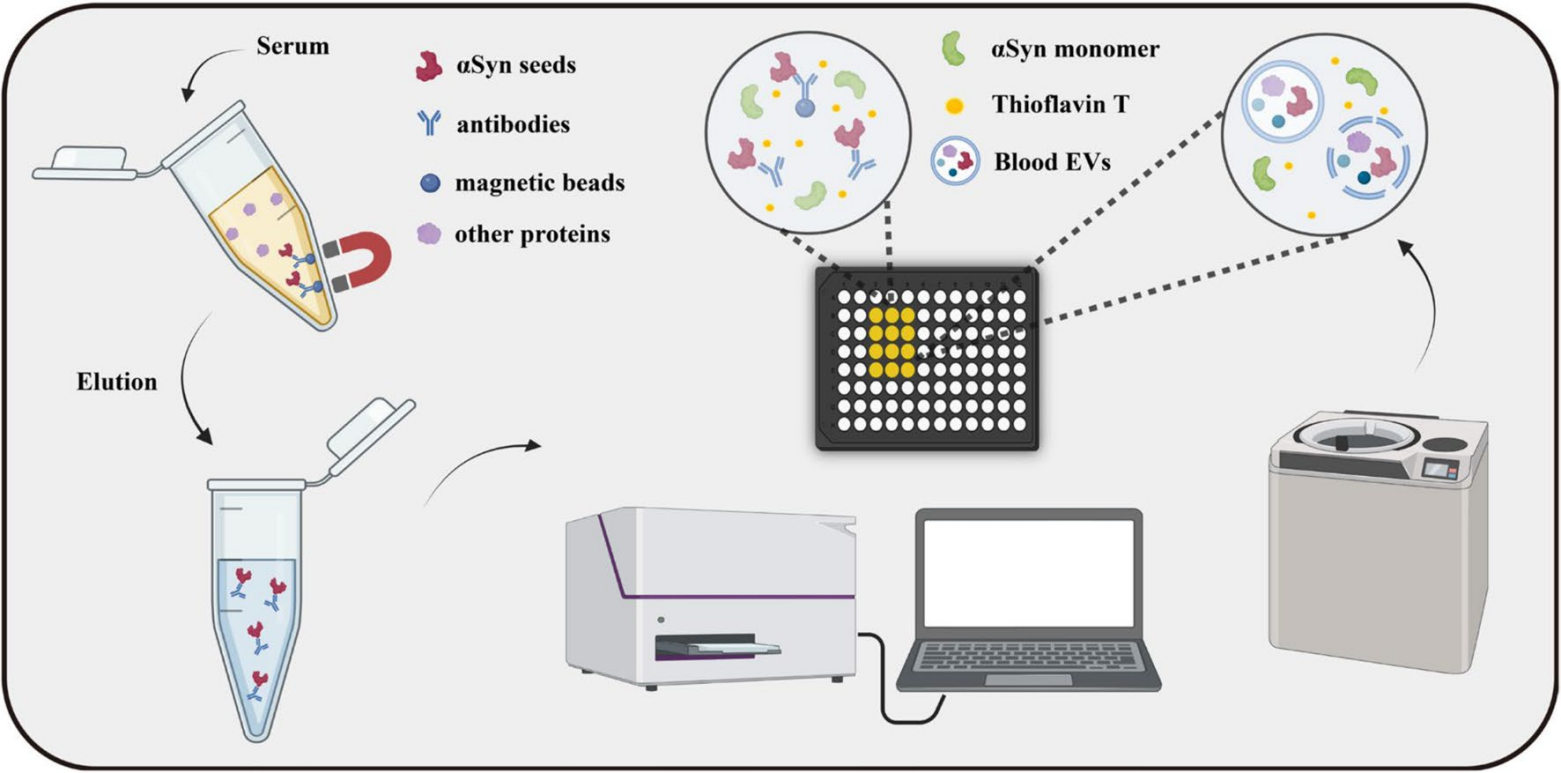
Steps of seed amplification assays (SAAs) involving immunoprecipitation (IP) and extracellular vesicles (EVs). αSyn in plasma can be isolated using magnetic beads coated with αSyn antibodies or by ultracentrifugation to separate EVs containing αSyn, followed by SAA. Graphic created with BioRender

However, detecting pathological αSyn in the blood is challenging due to its typically low concentration compared to CSF where αSyn levels reflect neuronal and glial activities, EV release, and contributions from peripheral tissues. In CSF, αSyn concentration averages around 1.36 ± 0.35 ng/ml, but in the serum, αSyn seeds are present at much lower concentrations [85]. Additionally, many proteins and substances in the blood can interfere with αSyn aggregation in vitro. For example, lipoproteins and serum albumin are known to inhibit αSyn aggregation, making the development of reliable blood assays for αSyn a complex task [86, 87]. Some recent serum assays have employed methods such as EV extraction or immunoprecipitation to remove these inhibitory components, facilitating the amplification of pathological αSyn seeds using SAA (Fig. 3). However, these techniques are time-consuming and not yet practical for large-scale use. Simplifying the process to amplify pathological proteins in serum is a promising area for future research. Before such an assay can be fully developed, technical challenges need to be addressed. These include optimizing the sample volume, preserving maximum seeding activity while removing inhibitory proteins, and concentrating amyloid fibrils from large serum samples. One potential method is the use of sarkosyl precipitation and ultracentrifugation, which isolate insoluble protein aggregates from biological samples [88]. This process reduces the concentrations of inhibitors in the blood, allowing pathological αSyn seeds to be detected without interference. Detecting pathological αSyn in the blood through SAA may eventually become feasible with optimization of the amplification process.

## Current optimization directions for αSyn SAAs

The sensitivity and specificity of SAAs for distinguishing various synucleinopathies from non-synucleinopathy controls are promising, but full validation is necessary before they can be implemented in clinical practice for diagnosing PD and other synucleinopathies. Several methodological variables—such as temperature, monomeric αSyn concentration, type of well plates, ionic strength and pH of reaction buffers, incubation times, detergent presence, and shaking protocols—can all impact the variability of results [65, 89–91]. Additionally, the composition and biological characteristics of the sample matrix and its dilution in the reaction mix are significant factors. Variations in protocols can lead to different αSyn conformations or tissue-specific amplifications, potentially altering assay performance. Multiple research groups are working to optimize assay conditions to improve detection limits and expand the range of biofluids and tissues that can be used. In the following, we will explore these challenges in greater detail, examining how protocol variations influence αSyn amplification and discussing strategies to address these issues.

A commonly used and well-characterized substrate for SAAs is full-length αSyn protein. However, recombinant αSyn from other mammals and mutant forms such as K23Q have been developed as monomer reservoirs to improve reaction conditions [65, 92, 93]. The K23Q mutant, known for its enhanced stability and amplification efficiency, is particularly notable [92]. Additionally, studies have shown seven distinct amino acid differences between mouse and human αSyn proteins, with the A53T mutation causing a “natively unfolded” structure that significantly affects the protein's behavior, resulting in a shorter lag phase in fibril formation compared to human wild-type and other mutant forms [94]. The concentration of αSyn monomers is also crucial. The Soto group's protocol utilized concentrations exceeding 1 mg/ml to ensure effective seed conversion and elongation [95]. Increasing the reaction temperature, typically ranging from 30 °C to 42 °C, in some cases even up to 50 °C to 70 °C, improves the assay efficiency by enhancing molecular motion [96, 97]. Shaking protocols with important parameters of intensity and duration, play a role in αSyn aggregation [98]. While neutral pH typically results in slow aggregation, vigorous shaking or the introduction of beads or surfactants can accelerate this process [99]. Lowering the pH to 5.5 can also significantly speed up aggregation, even without agitation, due to enhanced secondary nucleation at mildly acidic pH levels [100, 101]. The type of salt used in the reaction can also significantly influence amplification speed, with salts like SO₄^2^⁻ and Cl⁻ optimizing the difference between seeded and spontaneous fibrillization [102]. SO₄^2^⁻, in particular, facilitates critical interactions between proteins, water, and anions, promoting partial folding of αSyn and rapid amplification of oligomeric seeds [102, 103]. In some protocols, detergents like sodium dodecyl sulfate (SDS) are used, especially in CSF SAA protocols for detecting pathological proteins [98, 104]. SDS significantly accelerates αSyn aggregation, both with and without seeds. This effect has been documented in studies by Otzen et al., though they may introduce challenges in standardizing screening assays [98].

We propose several strategies to enhance the assay performance. While these techniques can significantly boost sensitivity and efficiency, they also have notable drawbacks. High monomer concentrations may cause non-specific aggregation due to increased protein density, leading to unwanted interactions [105]. Elevated temperatures, though effective for speeding up aggregation, can induce non-specific aggregation due to thermal instability or changes in protein dynamics [106]. Similarly, the use of beads and increased ionic strength may improve aggregation efficiency, but they also risk non-specific interactions, potentially leading to false positives or misleading results. Non-specific aggregation is a critical issue, as it can obscure true protein interactions and complicate data interpretation. Therefore, despite their advantages, these strategies must be carefully optimized and controlled to minimize their impact on specificity and ensure accurate results.

## Quiescent SAA

Building on traditional SAA principles, we have developed αSyn quiescent SAA (QSAA) through four key modifications of SAA [97]: raising the incubation temperature to 70 °C; utilizing a quiescent incubation mode; using mouse αSyn monomers instead of human αSyn monomers; and adding 10% ammonium sulfate to the incubation buffer. Unlike traditional methods which require agitation or sonication to promote aggregation, QSAA relies solely on a temperature-controlled fluorescence reader. This innovative technique facilitates the on-site amplification of αSyn seeds within brain homogenates and tissue sections. Mechanistically, the prion-like seeding activity of misfolded αSyn makes them as seeds to catalyze the transformation of soluble αSyn monomers into further misfolded aggregates, without any need of subsequent fragmentation (Fig. 2).

A key advantage of QSAA is the quiescent conditions, unlike physical agitation in other assays. By avoiding agitation, QSAA preserves the structural integrity of samples and prevents artificial fragmentation of the αSyn aggregates, providing precise and detailed information on both the distribution and the density of αSyn aggregates. Key differences between SAA and QSAA are summarized in Table 3.

**Table 3:** Comparison of performance between SAA and QSAA

	SAA	QSAA		QSAA in situ
Incubation mode	Sonication/shaking	Quiescent	Quiescent	
Cyclic	Cyclic fragmentation	One-step amplification	One-step amplification	
Detection device	Fluorescence-plate-reader	Real-time PCR instruments		Incubator
Reaction vessel	96-well plate	96-well PCR plate		24-well plate
Substrate type	Human αSyn (WT/mutant) monomer	Mouse αSyn monomer	Mouse αSyn monomer	
Substrate concentration	0.1–1.0 mg/ml	1.0 mg/ml	1.0 mg/ml	
AS addition	None	10% *w*/*v* AS	10% *w*/*v* AS	
Beads addition	Silicon/zirconia beads	None	None	
Reaction temperature	30–42 ℃	70 ℃	70 ℃	
Sample type	Liquid	Liquid		Slices
Incubation duration	24–120 h	6–24 h		6–24 h
Reaction volume	100 μl/well	20 μl/well		200 μl/well
Oil seal	None	Paraffin oil	Paraffin oil	
Fluorescent dye	ThT	ThT	ThT	
ThT concentration	5 μM	30 μM	30 μM	
Quantitative data	Lag phase/F_max_	Lag phase/F_max_		Fluorescence graph
Detection limit (PFFs)	Attograms	Femtograms	Femtograms	
Amplification mechanism	Nucleation, elongation	Secondary nucleation, elongation	Secondary nucleation, elongation	

SAA, seed aggregation assay; QSAA, quiescent aeed aggregation assay; AS, ammonium sulfate

QSAA has demonstrated exceptional sensitivity and specificity, both exceeding 90% in distinguishing between PD and non-PD cases across brain and skin tissue sections. It also correlates αSyn seeding activity with the spatial distribution of pathological αSyn in biological specimens. This highly sensitive and reliable assay offers the potential for deeper spatial insights into the pathological attributes of misfolded proteins within tissue Sects [97].

As a variant of SAA, QSAA has demonstrated high sensitivity in detecting pathological αSyn aggregates through a mechanism distinct from pS129 staining [107–110]. This suggests that QSAA could offer a reliable and comprehensive approach to studying the pathology of LBD. One key distinction between QSAA and pS129 staining lies in the timing and the nature of the markers they detect. While pS129 staining identifies phosphorylated αSyn, a marker that emerges after the initial deposition of the protein, QSAA targets the misfolded αSyn aggregates themselves, which likely form earlier in the disease process [39]. Importantly, pS129 is believed to inhibit the formation of seeded fibrils, meaning that by the time it becomes detectable, critical steps in pathological propagation may already have occurred [39]. This temporal difference highlights QSAA’s potential for earlier and accurate detection of disease progression.

## New Parkinson’s classification proposed based on biomarkers: two framework focuses on the biology of LBD

The pathological processes underlying PD begin many years before symptoms appear, by which time approximately 50% − 80% of dopamine-producing nigrostriatal cells are already lost [4]. This extensive neuronal loss poses significant challenges to the effectiveness of future disease-modifying interventions. To improve early diagnosis of synucleinopathies, two articles published in *The Lancet Neurology* presented distinct but complementary frameworks for biological definition of LBD. These frameworks aim to create a biological foundation for rigorous testing of research theories and ultimately aid in earlier diagnosis and intervention.

The first framework, the “Neuronal αSyn Disease Integrated Staging System (NSD-ISS)”, was developed by the research team led by Drs. Tanya Simuni and Ken Marek [14]. This system provides a biological definition of PD and DLB, introducing a schema for disease symptom progression. NSD-ISS is enabled by advances in αSyn SAA, which allows precise identification of pathological αSyn in CSF, providing reliable evidence for diagnosing synucleinopathies. Additionally, molecular imaging techniques such as dopamine transporter scans, neuromelanin-sensitive MRI, and single-photon emission computed tomography are recommended for quantifying the loss of dopaminergic neurons and confirming neurodegeneration in specific brain regions.

NSD-ISS enables researchers to study PD and DLB as a unified disease entity under the category of synucleinopathies, using three biological markers: neuronal αSyn (S), dopaminergic neuron dysfunction (D), and genetic status (G). These markers serve as anchors for staging the disease. Stages 1 and 2 are defined by S and D, while stages 3–6 are determined by combining biomarkers with clinical symptoms. However, NSD-ISS does not cover all PD and DLB cases. For instance, some individuals with inherited forms of PD may not exhibit pathological αSyn through SAA testing, meaning they would not fit within the NSD-ISS framework.

In parallel, a second framework, known as the “SynNeurGe Research Diagnostic Criteria”, was developed by Drs. Günter Höglinger and Anthony Lang [15]. This system also integrates three key biomarkers: pathological αSyn (S) in tissues or CSF, neuronal degeneration (N) as assessed through neuroimaging, and genetic variants (G) that cause or predispose individuals to PD. Unlike NSD-ISS, SynNeurGe incorporates the evaluation of pathological αSyn in skin and other biological materials as part of its diagnostic criteria, rather than being limited to CSF testing. It emphasizes the utility of αSyn SAA in skin samples, while also recommending immunohistochemistry or immunofluorescence techniques to detect αSyn, though these methods are less sensitive than skin SAA.

Both NSD-ISS and SynNeurGe are intended for research and clinical trials rather than for routine clinical diagnosis. These frameworks highlight the cumulative genetic risks, presence of pathological αSyn, and loss of dopaminergic neurons, aiming to create a biological foundation for understanding disease progression before the onset of parkinsonism. Both frameworks also employ SAA for highly sensitive detection of misfolded αSyn.

Despite their similarities, there are notable differences between the two frameworks: NSD-ISS introduces a staging system that includes functional impairment, making it particularly useful for early interventional trials. It emphasizes neuronal pathological αSyn and unifies PD and DLB under the term “neuronal αSyn disease”. SynNeurGe takes a novel approach by integrating the assessment of pathological αSyn in various tissues, including skin, which increases its practical applicability. However, it also includes cases where synucleinopathy is not identified, posing a potential risk for misclassification. The characteristics and differences between the NSD-ISS and SynNeurGe Research Diagnostic Criteria are summarized in Table 4.

**Table 4:** The characteristics and differences of the NSD-ISS and the SynNeurGe research criteria

	NSD-ISS	SynNeurGe
Purpose	Biological definition of disease	Biological definition of disease
Classification system	Yes	Yes
Integrated staging system	Yes	No
Disease Label	Neuronal α-synuclein disease	Parkinson’s disease
Genetic variants considered	Yes	Yes
α-Synuclein pathology	Yes	Yes
CSF seed amplification assays	Yes	Yes
Other assays involved	No	Skin seed amplification assays, skin immunohistochemistry
Neuronal dysfunction/neurodegeneration	Yes	Yes
DAT scan	Yes	Yes
Other imaging modalities	No	[^18^F]fluorodeoxyglucose-PET, metaiodobenzylguanidine SPECT
Staging system	Yes	No
Clinical signs and symptoms usage	Not used for diagnosis; used to distinguish stages	Not used for diagnosis; provides a list of related signs and symptoms

**DAT,** Dopamine transporter; **SPECT,** single-photon emission computed tomography

These research initiatives represent a potential turning point in the design of future clinical trials. However, PD is a clinical-pathological entity characterized by significant heterogeneity and clinical complexity. While αSyn plays a key role in its pathophysiology, the diverse manifestations of the disease complicate efforts to create uniform diagnostic and therapeutic approaches.

## The role of αSyn-SAA in Alzheimer’s disease (AD)

AD is a complex neurodegenerative disorder primarily characterized by the accumulation of abnormal neuritic plaques and neurofibrillary tangles in the brain [111]. While these hallmark features define AD, the presence of additional brain pathologies, referred to as copathologies, is increasingly recognized as common [111–114]. Among these, αSyn pathology is particularly prevalent, and is observed in over half of AD cases, as confirmed by various autopsy studies [115, 116]. αSyn copathology has also been found in conditions like amyotrophic lateral sclerosis (ALS) [117]. This has led to the inclusion of αSyn-SAA in the revised criteria for AD diagnosis, acknowledging the relevance of synuclein biomarkers since AD often coexists with other pathologies in older adults [118].

One key aspect of the interaction between AD and αSyn pathology is the colocalization of tau and αSyn aggregates within nerve cells [119]. Research has demonstrated that αSyn can initiate tau aggregation, while tau can accelerate the fibrillization and spread of αSyn [120–122]. This bidirectional relationship not only drives the progression of both pathologies but also creates a more complex and severe clinical presentation. AD patients who also exhibit Lewy body pathology experience a more rapid cognitive decline and have higher mortality rates compared to those with pure AD [114, 123]. This suggests that αSyn pathology exacerbates the severity of AD, potentially leading to a more aggressive disease course.

Despite the significant implications of αSyn pathology in AD, traditional methods for detecting pathological αSyn in AD patients have yielded inconclusive results, limiting our understanding of its role. Recent advancements in SAA have addressed this diagnostic challenge, revealing that αSyn-SAA can effectively detect αSyn pathology even in non-Lewy body diagnoses [112–114]. More importantly, the presence of pathological αSyn in CSF has been linked to specific clinical features in AD patients [113]. Understanding the relationship between AD and αSyn pathology could pave the way for accurate predictions of the disease trajectory observed in clinical practice.

## αSyn SAAs for differential diagnosis of synucleinopathies

**Fig. 4:**
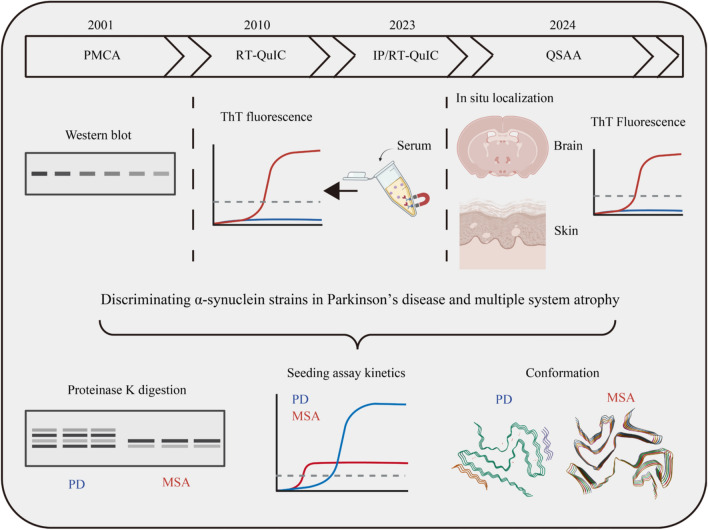
Evolution and applications of seed amplification assay (SAA) in differentiating between PD and MSA**.** Upper, outline of the history of SAA development; lower, three analytical methods: proteinase K digestion of the final products from the SAA, followed by Western blot detection; amplification kinetics analysis; and cryo-electron microscopy. These methods are utilized to differentiate between PD and MSA

The conformation and seeding behavior of pathological αSyn vary across neurodegenerative diseases, allowing for their differentiation through SAAs (Fig. 4). Research has shown that the seeding kinetics of αSyn aggregates differ between PD, MSA, and DLB, improving the accuracy of differential diagnosis. For instance, studies by Claudio Soto’s group, using CSF and postmortem brain samples from PD and MSA patients, identified faster aggregation kinetics in MSA-derived samples compared to PD [124]. However, despite this acceleration, MSA samples reached a lower fluorescence plateau than PD samples, indicating a more aggressive aggregation behavior in MSA. This plateau, which reflects beta-sheet structures in amyloid fibrils (indicated by Thioflavin T (ThT) fluorescence), suggests structural differences between MSA and PD aggregates. These structural variations have been validated by cryo-electron microscopy (cryo-EM)**,** which consistently shows that PD filaments have protofilament folds with eight beta-sheets**,** while MSA filaments have seven beta-sheets [125]. Interestingly, αSyn aggregates from different regions of the body show distinct aggregation behaviors. For instance, salivary samples from PD patients show faster aggregation kinetics than those from MSA, reflecting different disease progression in non-CNS tissues [126]. On the other hand, cutaneous samples from both PD and MSA display comparable kinetics, suggesting a more uniform αSyn strain in peripheral tissues [127]. αSyn aggregation kinetics have also been used to differentiate PD from DLB. Studies using CSF and postmortem brain samples indicate that DLB samples show faster aggregation and reach higher fluorescence maxima compared to PD samples, which can help distinguish between these two disorders [128].

The variability in diseases associated with αSyn has led to the “strains” hypothesis. According to this concept, the conformation of a misfolded protein determines its morphology**,** pathology, and functional properties, which in turn shape the disease phenotype [129]. Recent analyses using cryo-EM have revealed structural disparities in αSyn filaments from PD and MSA patients. In PD, the filaments tend to be elongated and linear with helical twists ranging from 76.6 to 199 nm, contributing to the formation of long, continuous fibrils. In contrast, MSA filaments display shorter helical twists**,** underscoring the unique molecular conformations associated with distinct synucleinopathies [124]. Recombinant αSyn monomers have been shown to aggregate into distinct forms with unique properties under varying conditions. Groundbreaking work by Bousset et al. highlighted this phenomenon by generating distinct conformations of aggregated wild-type αSyn in vitro [130]. By manipulating factors such as buffer composition and salinity, they generated two main forms: cylindrical structures termed “fibrils” and flat, twisting structures termed “ribbons”. These forms exhibited characteristic differences in seeding capacities**,** toxicity**,** inclusion formations**,** and dissemination pathways. Moreover, when elongated with monomeric αSyn, these structures maintained their original conformation, supporting the strain hypothesis [130, 131].

The structural diversity of αSyn strains across PD, MSA, and DLB is further reflected in their sensitivity to proteases and detergents**.** Studies have shown that αSyn from MSA samples is less stable in the presence of detergents compared to PD samples [132]. Despite this, αSyn aggregates from CSF samples of both PD and MSA patients exhibit high resistance to degradation [124, 133]. Under protease conditions, the N-terminal and middle regions of αSyn are protease-resistant, while the C-terminal region is fully degraded, suggesting that the C-terminal is not involved in aggregate formation. Moreover, under treatment with guanidine hydrochloride, a chaotropic agent, the MSA-derived αSyn is less stable than that from PD [133]. Similarly, SDS treatment resulted in more insoluble αSyn in DLB and PD samples compared to MSA [134]. The increased resistance of PD and DLB aggregates to detergents indicates a tighter packing of the aggregates, while the increased sensitivity of MSA aggregates to Proteinase K may be due to their rapid aggregation and looser structure, which could explain the faster progression observed in MSA.

In summary, the distinct structural and kinetic properties of αSyn aggregates offer critical insights into the differential diagnosis of synucleinopathies. Continued research is essential to unravel the complex interplay between αSyn conformation, aggregation dynamics, and disease progression, which will improve our ability to distinguish between PD, MSA, and DLB and develop targeted therapeutic approaches.

## Conclusions and future directions

αSyn SAAs have shown substantial potential in diagnosing synucleinopathies, particularly in early detection using CSF and other biological samples. While these assays have demonstrated effectiveness, they are insufficient for definitive diagnosis when used alone. Rather, αSyn SAAs should be integrated into a broader diagnostic approach that includes a variety of biomarkers, as exemplified by AD, where early biomarker identification has enabled pre-symptomatic interventions. However, identifying individuals before symptoms arise, though advantageous for early treatment, introduces ethical concerns, such as psychological impacts and potential stigmatization. These factors must be balanced carefully in clinical practice.

To further integrate αSyn SAAs into clinical use, several key challenges must be addressed. Standardized guidelines for sample collection, handling, and analysis are crucial to ensure consistent results across laboratories. Additionally, enhancing the sensitivity and specificity of the assay to detect early-stage pathological αSyn and accurately quantify its concentration is vital for monitoring disease progression and evaluating therapeutic responses.

Resolving these key issues will make αSyn SAAs a viable clinical tool for early and accurate diagnosis. When combined with other biomarkers—such as neurofilament light chain, amyloid, tau, and glial fibrillary acidic protein—and applied to diverse biological samples, these assays can significantly improve the diagnostic precision for synucleinopathies [135]. This holistic approach offers a promising path toward better disease management, early intervention, and development of personalized treatments for conditions like PD and related disorders. To ensure the success of this approach, ethical guidelines must also evolve, providing clarity on how to handle early detection and its societal implications, thus fostering a responsible and balanced application of these emerging technologies.

## Data Availability

Not applicable.

## Abbreviations

αSyn: α-Synuclein
SAAs: Seed amplification assays
QSAA: Quiescent seed amplification assay
PD: Parkinson's disease
MSA: Multiple system atrophy
DLB: Dementia with Lewy bodies
LBD: Lewy body diseases
NSD-ISS: Neuronal αSyn disease integrated staging system
EV: Extracellular vesicles
CNS: Central nervous system
CSF: Cerebrospinal fluid
ThT: Thioflavin T
NAC: Non-amyloid component
BBB: Blood-brain barrier
Hb-αSyn: Hemoglobin-binding α-synuclein
PrPSc: Scrapie prion protein
SDS: Sodium dodecyl sulfate
RT-QuIC: Real-time quaking-induced conversion
PMCA: Protein misfolding cyclic amplification
cryo-EM: Cryo-electron microscopy
